# Two New Bidesmoside Triterpenoid Saponins from the Seeds of *Momordica charantia* L.

**DOI:** 10.3390/molecules19022238

**Published:** 2014-02-21

**Authors:** Lin Ma, Ai-Hua Yu, Li-Li Sun, Wan Gao, Meng-Meng Zhang, Ya-Lun Su, Hua Liu, Tengfei Ji

**Affiliations:** 1 State Key Laboratory of Bioactive Substances and Functions of Natural Medicines, Institute of Materia Medica, Chinese Academy of Medical Sciences & Peking Union Medical College, Beijing 100050, China; 2 Jiangxi University of Traditional Chinese Medicine, Nanchang 330004, China

**Keywords:** *Momordica charantia* L., bidesmoside, triterpenoid saponin, NMR, MS

## Abstract

Two new bidesmoside triterpenoid saponins which were identifed as 28-*O*-*β*-d-xylopyranosyl(1→3)-*β*-d-xylopyranosyl(1→4)-*α*-l-rhamnopyranosyl(1→2)-[*α*-l-rhamno-pyranosyl(1→3)]-*β*-d-fucopyranosyl gypsogenin 3-*O*-*β*-d-glucopyranosyl (1→2)-*β*-d-glucopyranosiduronic acid (**C1**) and 28-*O*-*β*-d-xylopyranosyl(1→4)-*α*-l-rhamnopyranosyl (1→2)-[*α*-l-rhamnopyranosyl(1→3)]-*β*-d-fucopyranosyl gypsogenin 3-*O*-*β*-d-gluco-pyranosyl(1→2)-*β*-d-glucopyranosiduronic acid (**C2**) were isolated together with two known compounds from the seeds of *Momordica charantia* L. Their structures were elucidated by the combination of mass spectrometry (MS), one and two-dimensional NMR experiments and chemical reactions.

## 1. Introduction

The fruit, seeds, aerial parts and roots of *Momordica charantia* L. (Cucurbitaceae) have been used to treat diabetes. Over 100 compounds have been isolated from the fruits, seeds, leaves, canes and roots of this genus, mainly cucurbitane- and oleanene-type triterpenes, and recently studies have discovered that many new cucurbitane triterpenoids from the fruits and the roots of *M. charantia* L. [1,2,3], and cucurbitane triterpenoids from the fruits of this genus showed a significant enhancement of glucose disposal and increases in fatty acid oxidation. The cucurbitane triterpenoids from *M. charantia* L. may therefore provide novel leads for the development of a new class of AMPK-activating agents [4]. Five cucurbitane-type triterpene glycosides from the seeds of *M. charantia* L. was reported [5,6].

**Figure 1:**
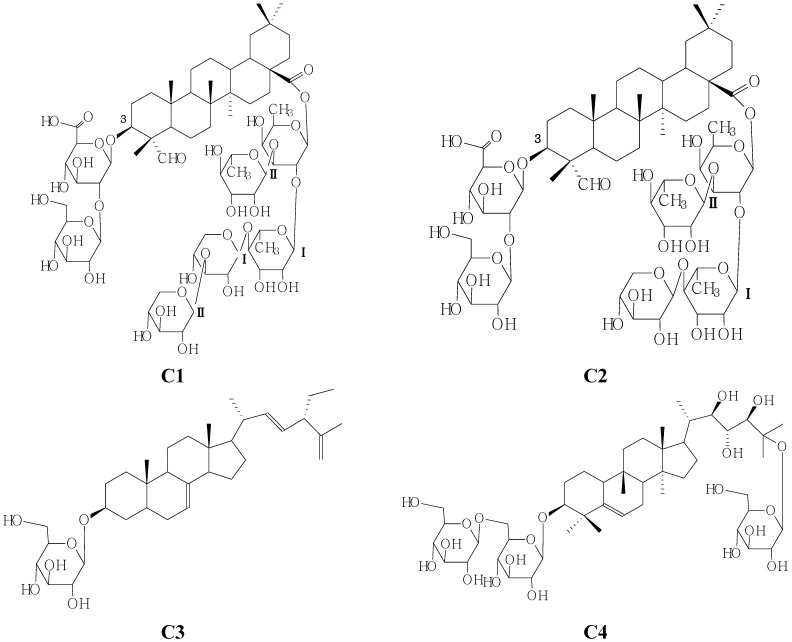
Structures of compounds **C1**–**4**.

In this paper, we now report the isolation and characterization of two new bidesmoside triterpenoid glycosides which were identified as 28-*O*-*β*-d-xylopyranosyl(1→3)-*β*-d-xylopyranosyl(1→4)-*α*-l-rhamnopyranosyl(1→2)-[*α*-l-rhamnopyranosyl(1→3)]-*β*-d-fucopyranosyl gypsogenin 3-*O*-*β*-d-glucopyranosyl (1→2)-*β*-d-glucopyranosiduronic acid (**C1**) and 28-*O*-*β*-d-xylopyranosyl(1→4)-*α*-l-rhamnopyranosyl(1→2)-[*α*-l-rhamnopyranosyl(1→3)]-*β*-d-fucopyranosyl gypsogenin 3-*O*-*β*-d-gluco-pyranosyl(1→2)-*β*-d-glucopyranosiduronic acid (**C2**) and two known compounds identified as 3-*O*-*β*-d-glucopyranosyl-24*β*-ethyl-5*α*-chole-sta-7,trans-22*E*,5(7)trien-3*β*-ol (**C3**) and momordicoside S(**C4**) (Figure 1) on the basis of spectroscopic and chemical evidence.

## 2. Results and Discussion

The *n*-BuOH-soluable portions of the 95% EtOH extract of seeds of *M. charantia* L. was subjected to silica gel, RP-18, and Sephadex LH-20 column chromatographies and semipreparative HPLC to yield two new oleanane-type triterpenoids **C1** and **C2** and two known compounds identified as 3-*O*-*β*-d-glucopyranosyl-24*β*-ethyl-5*α*-cholesta-7-*trans*-22*E*,5(7)trien-3*β*-ol (**C3**) and momordicoside S (**C4**).

Compound **C1** was an amorphous white powder, which gave a positive result in the Liebermann- Burchard test. Acid hydrolysis of compound **C1** with 2 mol/L HCl–1,4-dioxane (1:1, *v/v*) furnished d-xylose, l-rhamnose, d-glucose, and d-fucose in the ratio of 2:2:1:1 by HPLC analysis of the corresponding thiazolidine derivatives following conversion to the 1-[(*S*)-N-acetyl-(*R*)-methylbenzylamino]-1-deoxyalditol acetate derivatives [7]. In the (‒)- and (+)-ESI-MS of **C1**, quasimolecular ion peaks were observed at *m/z* 1,509 [M–H]^−^ and *m/z* 1,533 [M+Na]^+^ respectively, HR-ESI-MS (*m/z* 1,533.7190 [M+Na]^+^) analysis revealed the molecular formula of **C1** to be C_69_H_112_O_33_Na (calcd. 1,533.7168). The five fragment ions at *m/z* 1,377 [M–132–H], 1,329 [M–162–H], 1,171 [M–162–176–H], 807 [M–146 × 3–132 × 2–H] and 469 [M–146 × 3–132 × 2–162–176–H], indicated the sequential losses of seven sugar moieties (five hexoses and two pentoses).

The ^1^H (pyridine-*d*_5_) spectra of **C1** revealed the presence of nine methyl proton signals at *δ*_H_ 0.76 (Me-25), 0.89 (Me-29), 0.92 (Me-26), 0.96 (Me-30), 1.18 (Me-27), 1.36 (Me-24), 9.89 (H-29) and included signals due to a gyosogenin skeleton [8], 1.54, 3H, d, *J* = 5.5 Hz (Me-Fuc), 1.61, 3H, d, *J* = 5.5 Hz (Me-Rha**II**), 1.67, 3H, d, *J* = 5.5 Hz,(Me-Rha**I**), as well as an olefinic proton at δ_H_ 5.37, 1H, s (H-12); The signals at δ_C_ 122.4, 143.8 in the ^13^C-NMR spectrum were assigned to a 12(13)-ene grouping by comparison with literature data [5].

**Figure 2:**
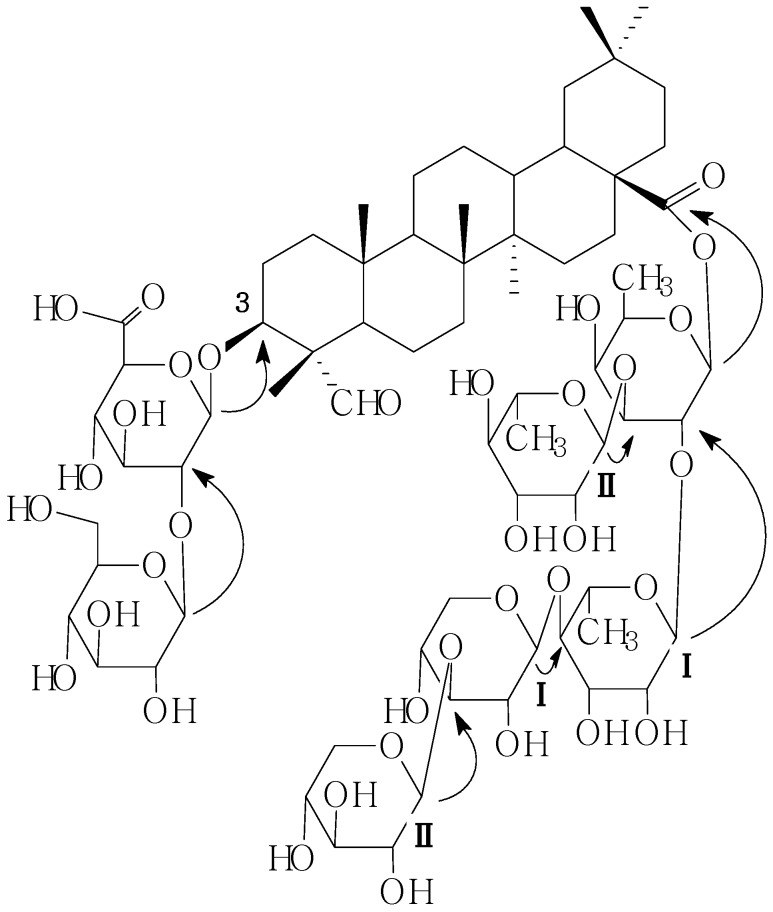
Key HMBC correlations of compound **C1**.

The ^1^H and ^13^C-NMR (Table 1) spectra of **C1** exhibited seven sugar anomeric protons assignable to a *β*-d-glucopyranosiduronic acid moiety [*δ*_H_ 4.76, 1H, d, 6.5 Hz], a *β*-d-glucopyranosyl moiety[*δ*_H_ 5.38, 1H, s], a *β*-d-fucopyranosyl moiety [*δ*_H_ 6.30, 1H, d, 5.5 Hz ], two *α*-l-rhamnopyranosyl moieties [*δ*_H_ 5.74, 1H, s (Rhm**I**-1-H); *δ*_H_ 5.67, 1H, s (Rhm**II**-1-H)], two *β*-d-xylopyranosyl moieties [*δ*_H_ 5.08, 1H, d, 7.0 Hz (Xyl**I**-1-H); *δ*_H_ 5.21, 1H, d, 7.0 Hz (Xyl**II**-1-H)], and a gyosogenin moiety [*δ*_H_ 3.04, 1H, dd-like, (18-H), *δ*_H_ 4.04, 1H, m (3-H), *δ*_H_ 5.44, 1H, s, (12-H); *δ*_H_ 9.89, 1H, s (23-H)]. The identities of the monosaccharides and the oligosaccharide sequence were determined by a combination of DEPT and two-dimensional NMR experiments (such as HMQC, HMBC, TOCSY NMR). The sequence of the glycan part was deduced from the following HMBC correlations: the anomeric proton signals at *δ*_H_ 4.76 and *δ*_C_ 83.5 (H-1 of the *β*-d-glucopyranosiduronic acid attached to the C-3 of the aglycone), *δ*_H_ 5.38 and *δ*_C_ 82.6 (H-1 of the *β*-d-glucopyranosyl moiety attached to the C-2 of the *β*-d-gluco-pyranosiduronic acid), and on the other hand, the pentasaccharide part at C-28 was established by the following HMBC information between the following protons and carbons: Fuc-1-H and 28-C, RhmI-1-H and Fuc-2-C, RhmII-1-H and Fuc-3-C, XylI-1-H and RhmI-4-C, XylII-1-H and XylI-3-C (Figure 2). On the basis of the foregoing evidence, the structure of **C1** was determined as 28-O-β-d-xylopyranosyl(1→3)-β-d-xylopyranosyl(1→4)-α-l-rhamnopyranosyl(1→2)-[α-l-rhamnopyranosyl-(1→3)]-β-d-fucopyranosylgypsogenin 3-O-β-d-glucopyranosyl (1→2)-β-d-glucopyranosiduronic acid.

**Table 1:** ^1^H-NMR (500 MHz) and ^13^C-NMR (125 MHz) data for **C1** in pyridine-*d*_5_ (δ, ppm).

Position	C1-Aglycone		Position	C1-Sugar Chain	
	^13^C	^1^H		^13^C	^1^H
1	39.8		GlcA-1	102.7	4.76, 1H, d, 6.5 Hz
2	25.8		2	82.6	4.32, 1H, m
3	83.5	4.04, 1H, m	3	77.8	4.65, 1H, m
4	54.7	------	4	72.4	4.20, 1H, m
5	48.1		5	76.9	4.50, 1H, m
6	18.7		6	175.9	
7	32.9		Glc-1	105.9	5.38, 1H, s
8	39.8		2	75.1	4.15, 1H, m
9	47.5		3	77.8	4.25, 1H, m
10	35.9		4	71.4	4.02, 1H, m
11	23.5		5	77.9	4.20, 1H, m
12	122.4	5.44, 1H, s	6	61.8	3.65, 2H, m
13	143.8	-------	Fuc-1	93.43	6.30, 1H, d, 5.5 Hz
14	41.7	------	2	76.7	4.25, 1H, m
15	27.9		3	83.7	3.95, 1H, m
16	23.5		4	72.4	3.86, 1H, m
17	46.7	-------	5	74.0	3.80, 1H, m
18	41.8	3.04, 1H, dd-like	6	18.3	1.54, 3H, d, 5.5 Hz
19	46.7		RhmI-1	100.9	5.74, 1H, s
20	30.6	------	2	71.4	4.33, 1H, m
21	32.1		3	72.3	4.28, 1H, m
22	32.9		4	82.9	4.50, 1H, m
23	209.7	9.89, 1H, s	5	68.7	4.01, H, m
24	10.9	1.36, 3H, s	6	18.3	1.67, 3H, d, 5.5 Hz
25	15.4	0.76, 3H, s	RhmII-1	101.2	5.67, 1H, s
26	17.3	0.92, 3H, s	2	71.4	3.26, 1H, m
27	26.1	1.18, 3H, s	3	72.1	4.32, 1H, m
28	175.9	------	4	70.6	4.71, 1H, m
29	32.1	0.89, 3H, s	5	68.7	4.51, 1H, m
30	23.5	0.96, 3H, s	6	18.3	1.61, 3H, d, 5.5 Hz
			XylI-1	106.2	5.08, 1H, d, 7.0 Hz
			2	74.8	4.01, 1H, m
			3	86.7	4.18, 1H, m
			4	70.2	4.13, 1H, m
			5	66.6	4.28/3.45, 2H, m
			XylII-1	105.6	5.21, 1H, d, 7.0 Hz
			2	76.3	4.04, 1H, m
			3	77.9	4.16, 1H, m
			4	70.6	4.13, 1H, m
			5	67.0	4.28/3. 61, 2H, m

Compound **C2** was obtained as a white, amorphous powder, In the (−)- and (+)-ESI-MS of **C2**, quasimolecular ion peaks were observed at *m/z* 1,377 [M-H]^−^ and, *m/z* 1,401 [M+Na]^+^, respectively, and HR-ESI-MS analysis revealed the molecular formula of **C2** to be C_65_H_102_O_31_ (calcd. 1401.6297, observed *m/z* 1,401.6345 [M+Na]^+^). Eight fragment ions at 1,247 [M–132+H], 1,217 [M–162+H], 1,085 [M–162–132+H], 939 [M–132–162–146+H], 909 [M–162–132–176+H], 793 [M–162–132–146–146+H], 647 [M–132–162–146–146–146+H] and 436 [M–132–162–146–146–146–176+H], indicated the sequential losses of one pentose and five hexoses. Acid hydrolysis of compound **C2** with 2 mol/L HCl–1,4-dioxane (1:1, *v/v*) furnished d-xylose, l-rhamnose, d-glucose, d-fucose in the ratio of 1:2:1:1 which were identified by HPLC analysis of the thiazolidine derivatives following conversion to the corresponding 1-[(*S*)-*N*-acetyl-(*R*)-methylbenzylamino]-1-deoxyalditol acetate derivatives [8].

The ^1^H-NMR spectrum of **C2** revealed the presence of nine methyl proton signals at *δ*_H_ 0.75 (Me-25), 0.88 (Me-29), 0.93 (Me-26), 0.95 (Me-30), 1.18 (Me-27), 1.37 (Me-24), 9.90 (H-29) and it included signals due to a gyosogenin skeleton [8], 1.53, 3H, d, *J* = 6.0 Hz (Me-Fuc), 1.62, 3H, d*,* *J* = 6.5Hz (Me-Rha**II**), 1.72, 3H, d, *J* = 6.0 Hz (Me-Rha**I**), as well as an olefinic proton at *δ*_H_ 5.37, 1H, s (H-12); The signals at *δ*_C_ 122.5, 143.9 in the ^13^C-NMR spectrum were assigned to the 12(13)-ene bond by comparison with literature data [5].

**Figure 3:**
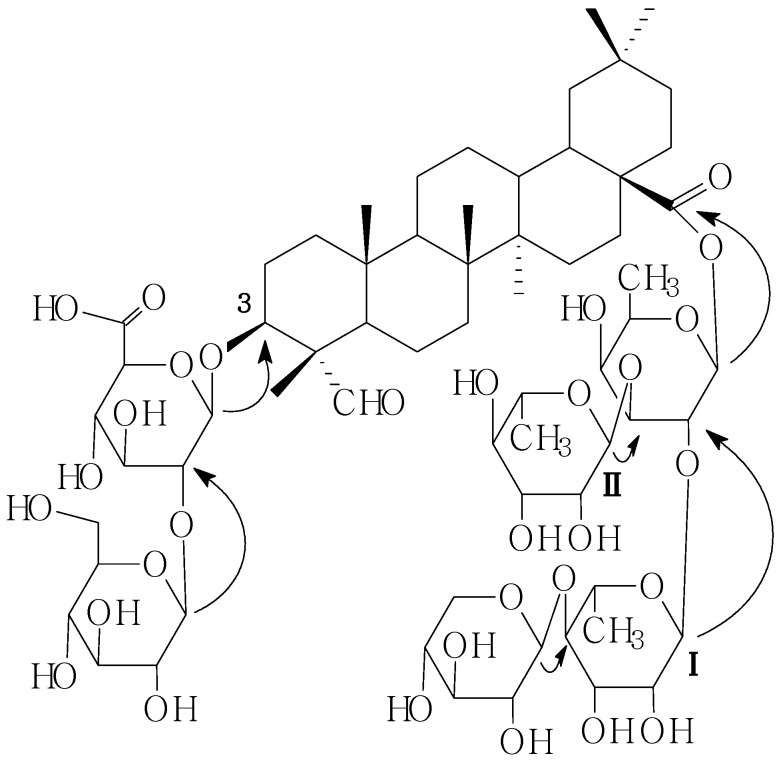
Key HMBC correlations of compound **C2**.

Comparison of the ^1^H-NMR and ^13^C-NMR spectral data of compound **C2** (Table 2) with that of **C1** indicated the compound **C2** lacked the xylpyranosyl moiety of **C1**. The identities of the monosaccharides and the oligosaccharide sequence were determined by a combination of DEPT and two-dimensional NMR experiments (such as HMQC, HMBC, TOCSY NMR). The sequence of the glycan part was deduced from the following HMBC correlations: the anomeric proton signals at *δ*_H_ 4.77 and *δ*_C_ 83.9 (H-1 of the *β*-d-glucopyranosiduronic acid attached to the C-3 of the aglycone), *δ*_H_ 5.37 and *δ*_C_ 82.2 (H-1 of the *β*-d-glucopyranosyl moiety attached to the C-2 of the *β*-d-gluco-pyranosiduronic acid), and on the other hand, the oligosaccharide part at C-28 was established by the HMBC correlationns between the following protons and carbons: Fuc-1-H and 28-C, Rhm**I**-1-H and Fuc-2-C, Rhm**II**-1-H and Fuc-3-C, Xyl-1-H and Rhm**I**-4-C (Figure 3). On the basis of the foregoing evidence, the structure of **C2** was established as 28-*O*-*β*-d-xylopyranosyl(1→4)-*α*-l-rhamno-pyranosyl(1→2)-[*α*-l-rhamnopyranosyl(1→3)]-*β*-d-fucopyranosyl gypsogenin 3-*O*-*β*-d-gluco-pyranosyl(1→2)-*β*-d-glucopyranosiduronic acid.

**Table 2:** ^1^H-NMR (500 MHz) and ^13^C-NMR (125 MHz) data for **C2** in pyridine-*d*_5_ (δ, ppm).

Position	C2-Aglycone		Position	C2-Sugar Chain	
	^13^C	^1^H		^13^C	^1^H
1	38.0		GlcA-1	102.9	4.77, 1H, brs
2	25.9		2	82.2	4.36, 1H, m
3	83.9	4.04, 1H, m	3	77.8	4.65, 1H, m
4	54.9	------	4	72.4	4.20, 1H, m
5	48.2		5	76.9	4.54, 1H, m
6	18.6		6	175.6	
7	32.4		Glc-1	106.2	5.37, 1H, s
8	40.0		2	75.4	4.51, 1H, m
9	47.6		3	78.4	4.52, 1H, m
10	35.6		4	71.6	4.06, 1H, m
11	23.6		5	77.9	4.36, 1H, m
12	122.5	5.37, 1H, s	6	62.0	4.57, 2H, m
13	143.9	-------	Fuc-1	93.6	6.29, 1H, d, 5.5 Hz
14	41.8	------	2	76.5	3.99, 1H, m
15	28.0		3	83.3	4.31, 1H, m
16	23.7		4	72.3	4.05, 1H, m
17	46.9	-------	5	74.2	4.07, 1H, m
18	42.0	3.10, 1H, dd-like	6	18.4	1.53,3H, d, 6.0 Hz
19	46.1		RhmI-1	100.8	5.77, 1H, s
20	30.7	------	2	70.8	4.33, 1H, m
21	32.2		3	72.4	4.28, 1H, m
22	33.0		4	83.0	4.52, 1H, m
23	209.8	9.90, 1H, s	5	68.7	3.13, 1H, m
24	10.9	1.37, 3H, s	6	18.8	1.72, 3H, d, 6.0 Hz
25	15.6	0.76, 3H, s	RhmII-1	101.5	5.68, 1H, s
26	17.4	0.93, 3H, s	2	71.6	3.85, 1H, m
27	25.9	1.18, 3H, s	3	72.4	4.32, 1H, m
28	176.2	------	4	70.4	4.75, 1H, m
29	32.0	0.88, 3H, s	5	68.7	4.53, 1H, m
30	23.1	0.95, 3H, s	6	18.6	1.62, 3H, d, 6.5 Hz
			Xyl-1	106.9	5.15, 1H, d,7.0 Hz
			2	74.8	4.04, 1H, m
			3	86.9	4.21, 1H, m
			4	70.0	4.13, 1H, m
			5	67.3	4.27/3.48, 2H, m

## 3. Experimental Section

### 3.1. General

Melting points were determined of an XT_4_-100_x_ micromelting apparatus (Beijing Keyi Electric Light Instrument Factory, Beijing, China) and are uncorrected. Optical rotations were measured with a Perkin-Elmer 241 MC polarimeter (PerkinElmer Inc., Waltham, MA, USA). IR spectra were obtained of Nicolet 5700 IR spectrometer (Thermo Fisher Scientific, Inc., Waltham, MA, USA). NMR spectra were recorded on an Inova 500 (^1^H, 500 MHz; ^13^C, 125 MHz) spectrometer (Agilent Technologies, Inc., Santa Clara, CA, USA). ESI-MS was performed with Agilent 1100 LC/MSD (Agilent Technologies, Inc., Santa Clara, CA, USA). For column chromatography, silica gel (200-300 mesh, Qingdao Marine Chemical Inc., Qingdao, China), ODS (40–60 μm, Alltech), and Sephadex LH-20 (Pharmacia Biotech AB, Uppsala, Sweden) were used. The analytical HPLC was performed on an Agilent 1200 LC equipped with a DAD and the preparative HPLC was performed on a Shimadzu LC-20A (Shimadzu LC-20A, Kyoto, Japan) equipped with a YMC-Pack ODS column (20 × 250 mm, 10 μm, YMC Co. Ltd. Kyoto, Japan).

### 3.2. Plant Material

The seeds of *Momordica charantia* L. were purchased from Anguo of Hebei Province in 2011, and identified by Lin Ma at Institute of Materia Medica, Chinese Academy of Medical Sciences & Peking Union Medical College. A voucher specimen has been deposited at our lab at the Institute of Materia Medica, Chinese Academy of Medical Sciences & Peking Union Medical College.

### 3.3. Extraction and Isolation

The seeds of *M. charantia* L. (15.0 kg) were defatted three times by petroleun ether (90 L each time), and then extracted three times by heating to reflux with 95% ethanol (90 L each time), and the combined solution was concentrated under reduced pressure to yield an extract (1.6 kg). The alcohol extract was partitioned successively with CHCl_3_, EtOAc and *n*-BuOH. The *n*-BuOH-soluble portions (175 g) was subjected to normal phase silica gel column chromatography with gradient elution [CHCl_3_–MeOH 20:1 (2 L), CHCl_3_–MeOH 9:1 (2 L), CHCl_3_–MeOH 4:1 (2 L), CHCl_3_–MeOH–H_2_O 7:3:0.5 (2 L), CHCl_3_–MeOH–H_2_O 6:4:0.5 (2 L), MeOH (2 L)] to give eleven fractions (Fr1-11). Compound C1 (8.0 mg) was purified from Fr9 (7.6 g) by normal-phase silica gel and Sephadex LH-20 chromatography. Compound C2 (5.0 mg) was purified from Fr10 (20.6 g) by repeated column chromatography as well as compound C1. Compound C3 (210.0 mg) was purified from Fr10 (20.6 g) by repeated column chromatography over silica gel with eluent as CHCl_3_–MeOH–H_2_O 4:1:0.1. Compound C4 (102.0 mg) was purified from Fr11 (12.5 g) by repeated column chromatography over silica gel with eluent as CHCl_3_–MeOH–H_2_O 6:4:0.5, and further purified by Sephadex LH-20 with eluent as MeOH and preparative HPLC (MeOH–H_2_O, 60:40). 

### 3.4. Acid Hydrolysis of Compounds C1 and C2

d-Glucose, d-galactose, l-rhamnose, d-fucose, d-xylose and l-arabinose aqueous solution (each 2 mg/mL, 80 μL) PMP CH_3_OH solution (0.5 mol/L, 80 μL) and aqueous NaOH solution (0.3 mol/L, 80 μL) were heated at 70 °C for 30 min, cooled to room temperature for 10 min, HCl aqueous solution added (0.3 mol/L, 80 μL) and extracted with CHCl_3_ (0.5 mL, three times). The aqueous fractions were identified by HPLC analysis (Phenomenex C18, 250 mm × 4.6 mm, 5 μm column); flow phase A: CH_3_CN-20 mmol/L NH_4_OAc aqueous solution (15:85), B: CH_3_CN-20 mmol/L NH_4_OAc aqueous solution (40:60), flow rate: 1.2 mL/min; gradient elution, 0→20 min, volume fraction of B from 0 to 60%; detection wavelengths: 245 nm; sample volume: 20 μL).

Compounds C1 (2 mg) and C2 (2 mg) were heated in an ampule with aqueous 2 mol/L HCl–1,4-dioxane (1:1, 2 mL) at 80 °C for 6 h. The aglycon was extracted with chloroform, and the aqueous layer was evaporated under reduced pressure and subjected to the normal preparation of sugar derivatives. Thus, compound C1 furnished d-xylose, l-rhamnose, d-glucose, and d-fucose in a ratio of 2:2:1:1, and compound C2 furnished d-xylose, l-rhamnose, d-glucose and d-fucose in the ratio of 1:2:1:1, which were identified by HPLC analysis of the thiazolidine derivatives following conversion to the 1-[(*S*)-*N*-acetyl-(*R*)-methylbenzylamino]-1-deoxyalditol acetate derivatives [7]. 

## 4. Conclusions

Two new bidesmoside triterpenoid glycosides identified as 28-*O*-*β*-d-xylopyranosyl-(1→3)-*β*-d-xylopyranosyl(1→4)-*α*-l-rhamnopyranosyl(1→2)-[*α*-l-rhamnopyranosyl(1→3)]-*β*-d-fucopyranosyl gypsogenin 3-*O*-*β*-d-glucopyranosyl (1→2)-*β*-d-glucopyranosiduronic acid (**C1**) and 28-*O*-*β*-d-xylopyranosyl(1→4)-*α*-l-rhamnopyranosyl(1→2)-[*α*-l-rhamnopyranosyl(1→3)]-*β*-d-fucopyranosyl gypsogenin 3-*O*-*β*-d-glucopyranosyl(1→2)-*β*-d-glucopyranosiduronic acid (**C2**) were isolated from the seeds of *M. charantia* L. together with two known ones identified as 3-*O*-*β*-d-glucopyranosyl-24*β*-ethyl-5*α*-cholesta-7-*trans*-22*E*,5(7)trien-3*β*-ol (**C3**) and momordicoside S (**C4**). This finding represents an addition to the ongoing research on the pharmacological activity of these compounds, which may be helpful to understand the use of this plant in traditional medicine and should continue to clarify its actual health benefits.

## Supplementary Materials

Supplementary materials can be accessed at: http://www.mdpi.com/1420-3049/19/2/2238/s1.
