# Supplementary material for: Two New Bidesmoside Triterpenoid Saponins from the Seeds of Momordica charantia L
Source: Molecules. 2014 Feb 21;19(2):2238–46. doi: 10.3390/molecules19022238 (PMC6271465; doi:10.3390/molecules19022238)
Supplement: Supplementary file 1 [file molecules-19-02238-s001.pdf]

# Supplementary Materials

**Figure S1.** The TOCSY Spectrum of Compound **C1**.

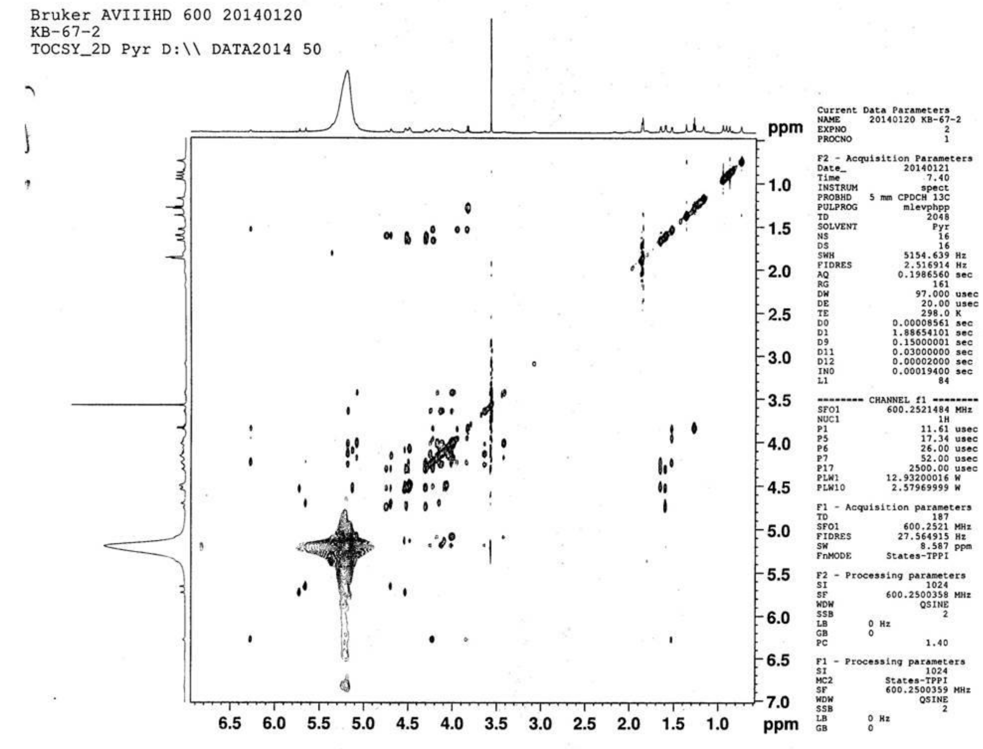


The whole TOCSY Spectrum of Compound C1.

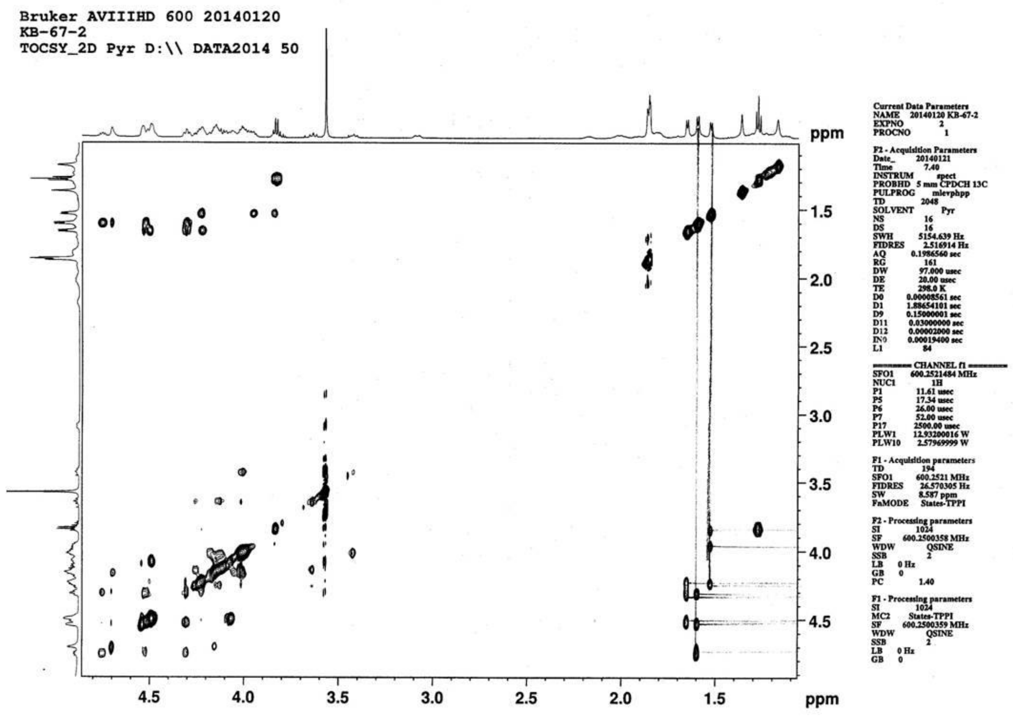


The part 1 of the TOCSY Spectrum of Compound C1.

**Figure S1.** *Cont.*

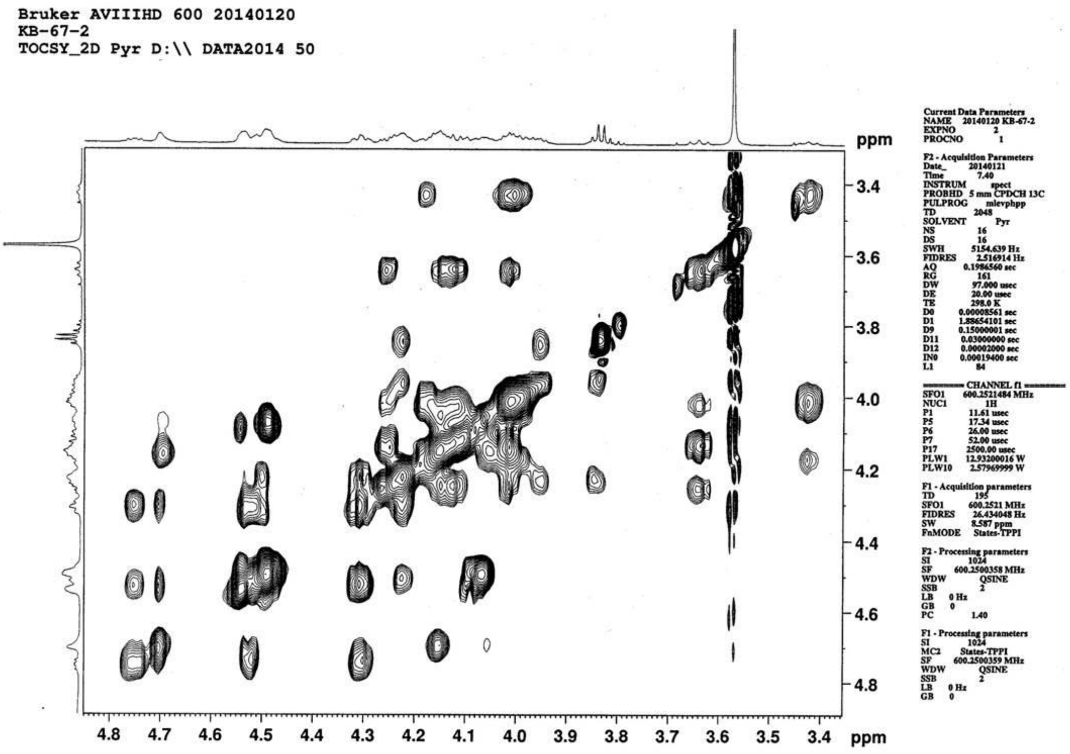


The part 2 of the TOCSY Spectrum of Compound C1.

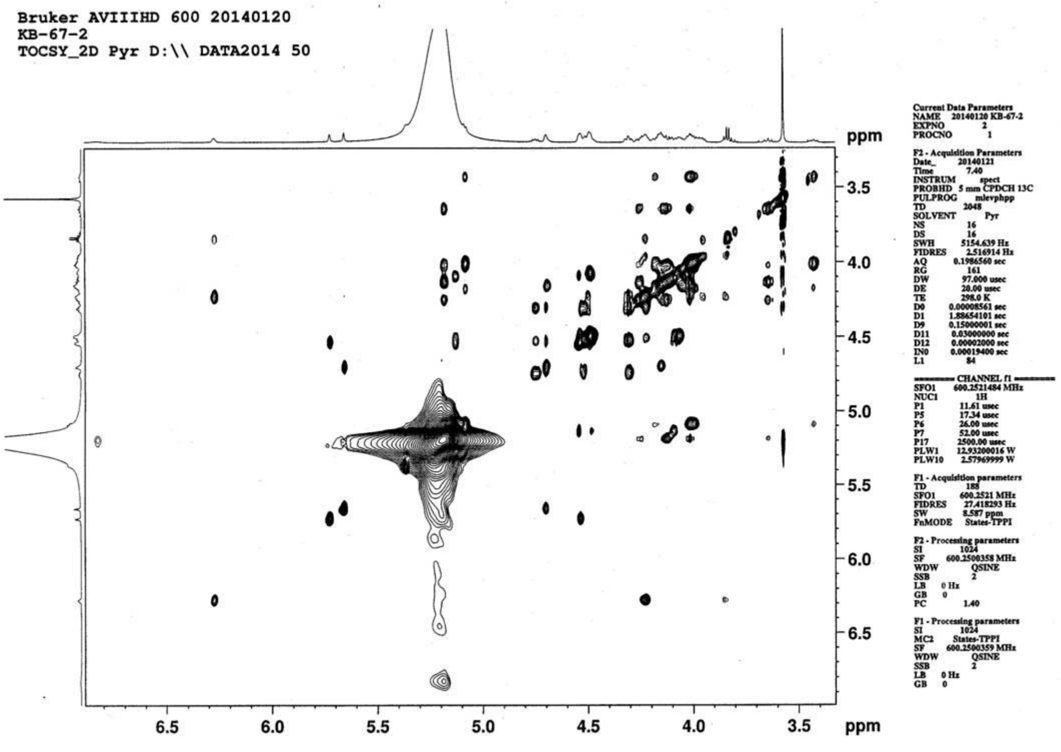


The part 3 of the TOCSY Spectrum of Compound C1.

**Figure S1.** *Cont.*

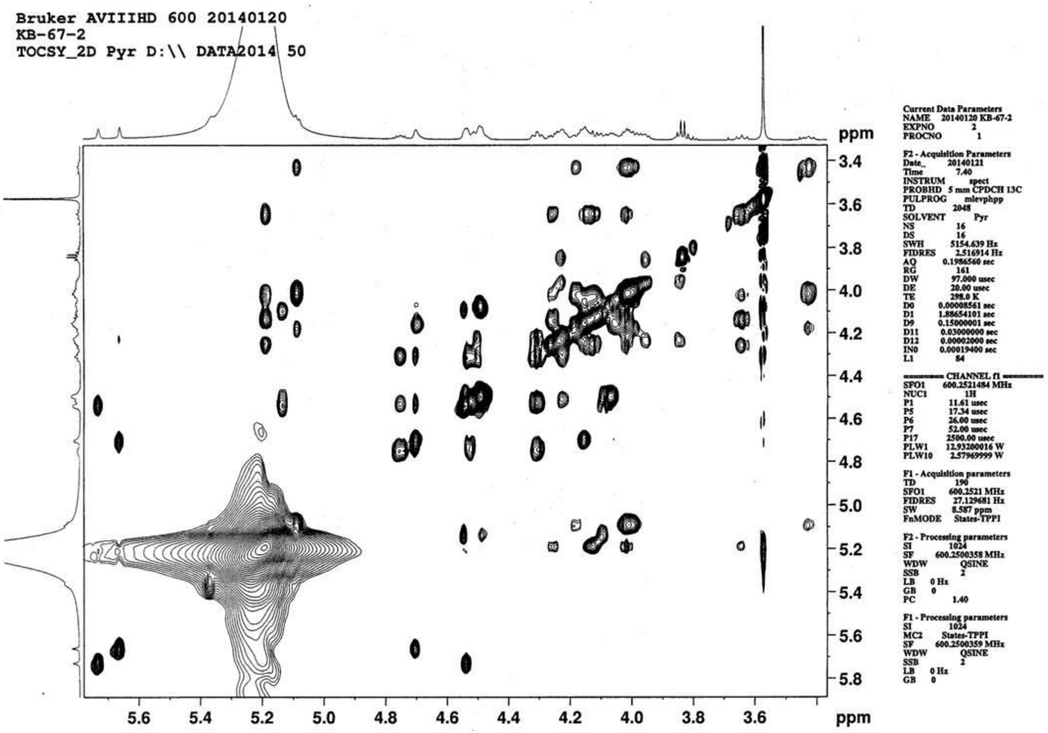


The part 4 of the TOCSY Spectrum of Compound C1.

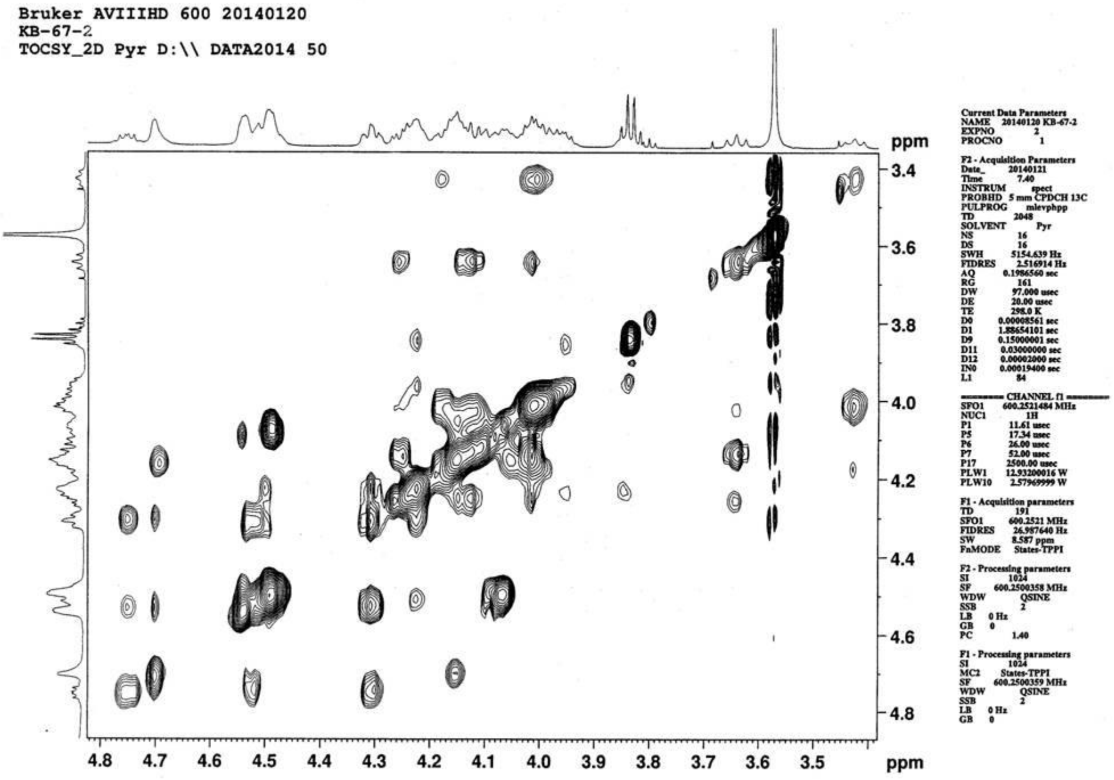


The part 5 of the TOCSY Spectrum of Compound C1.

**Figure S2.** The TOCSY Spectrum of Compound **C2.**

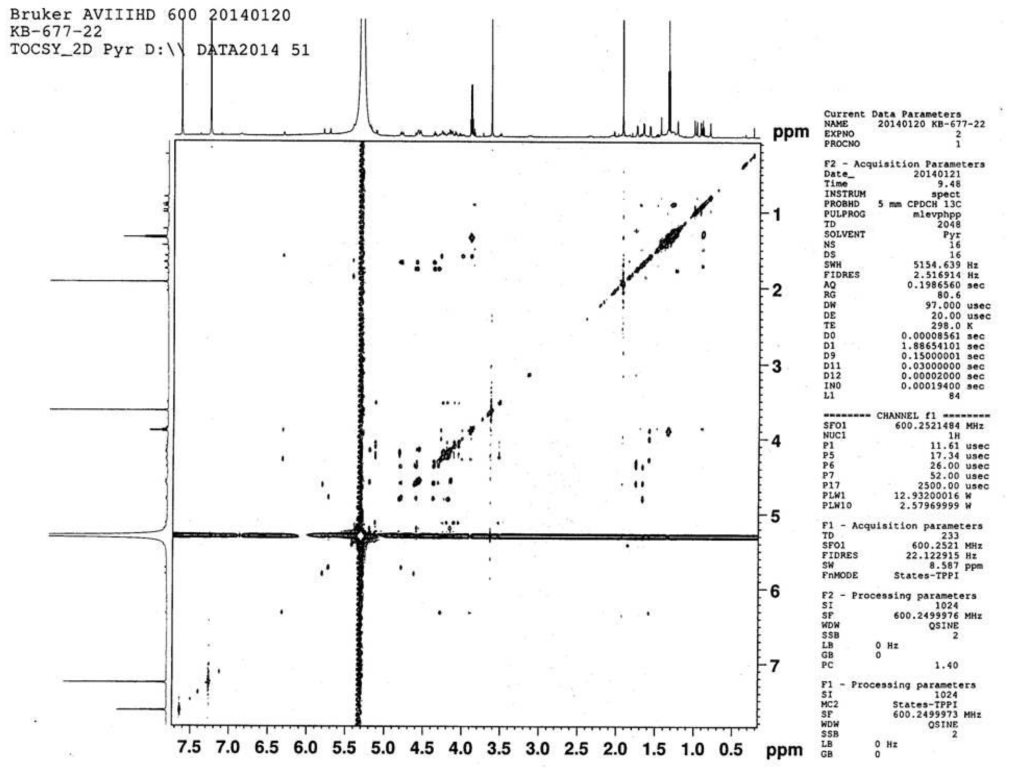


The whole TOCSY Spectrum of Compound C2.

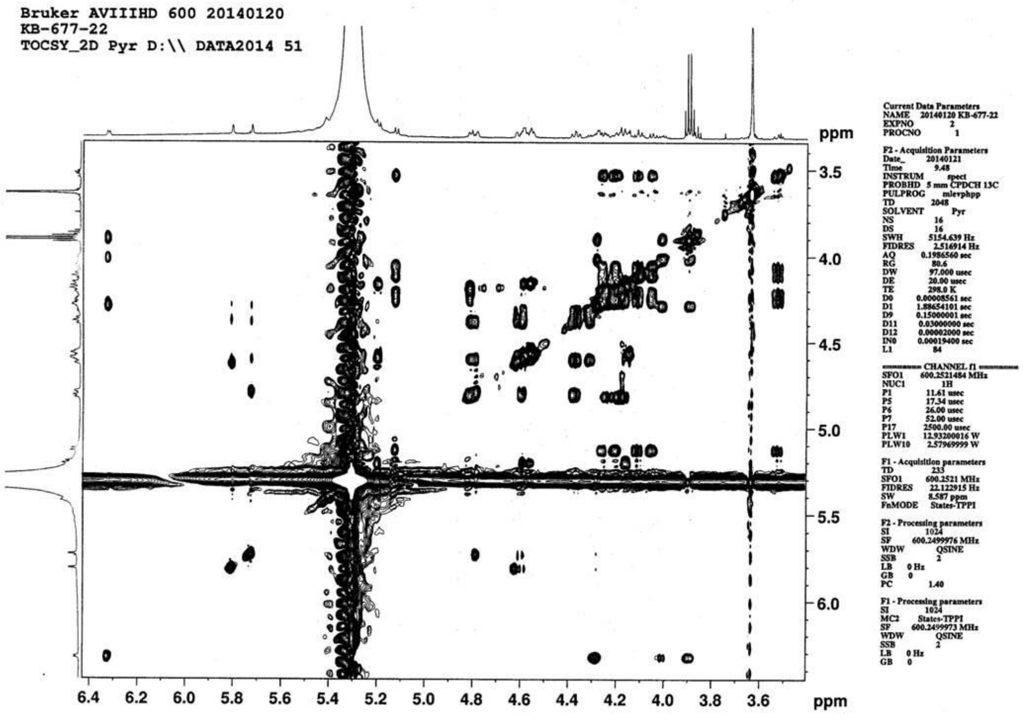


The part 1 of the TOCSY Spectrum of Compound C2.

**Figure S2.** *Cont.*

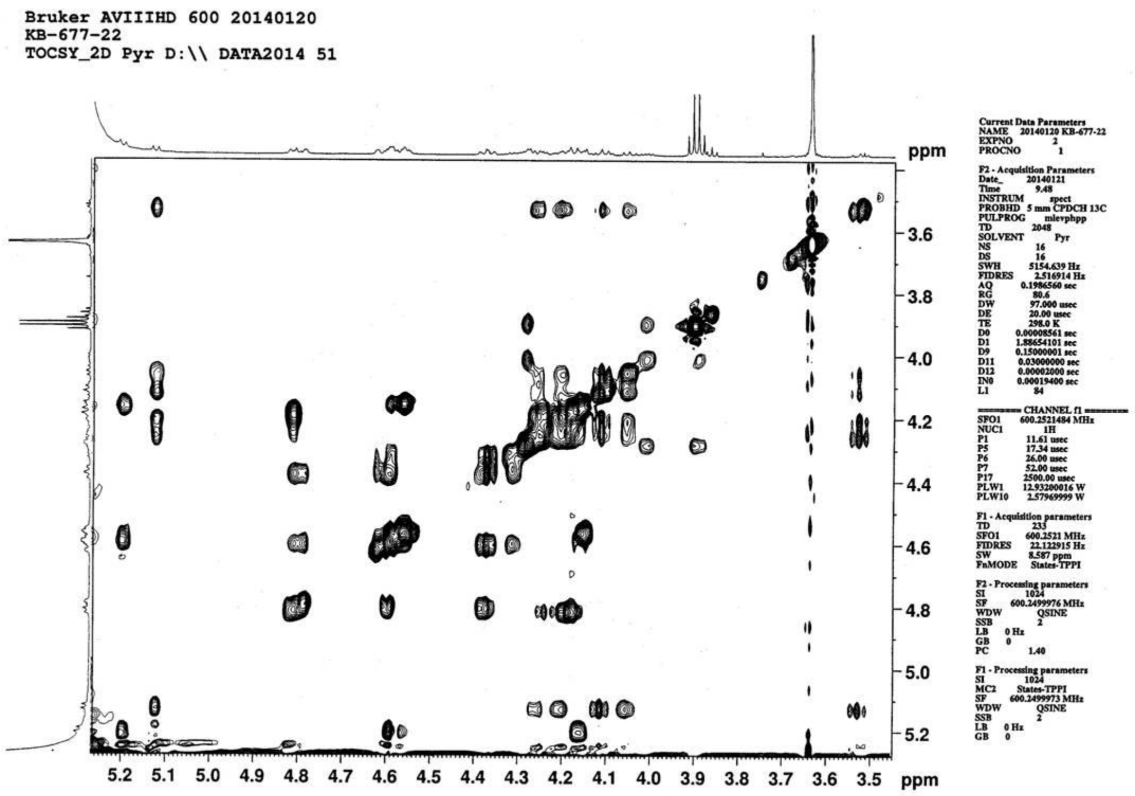


The part 2 of the TOCSY Spectrum of Compound C2.

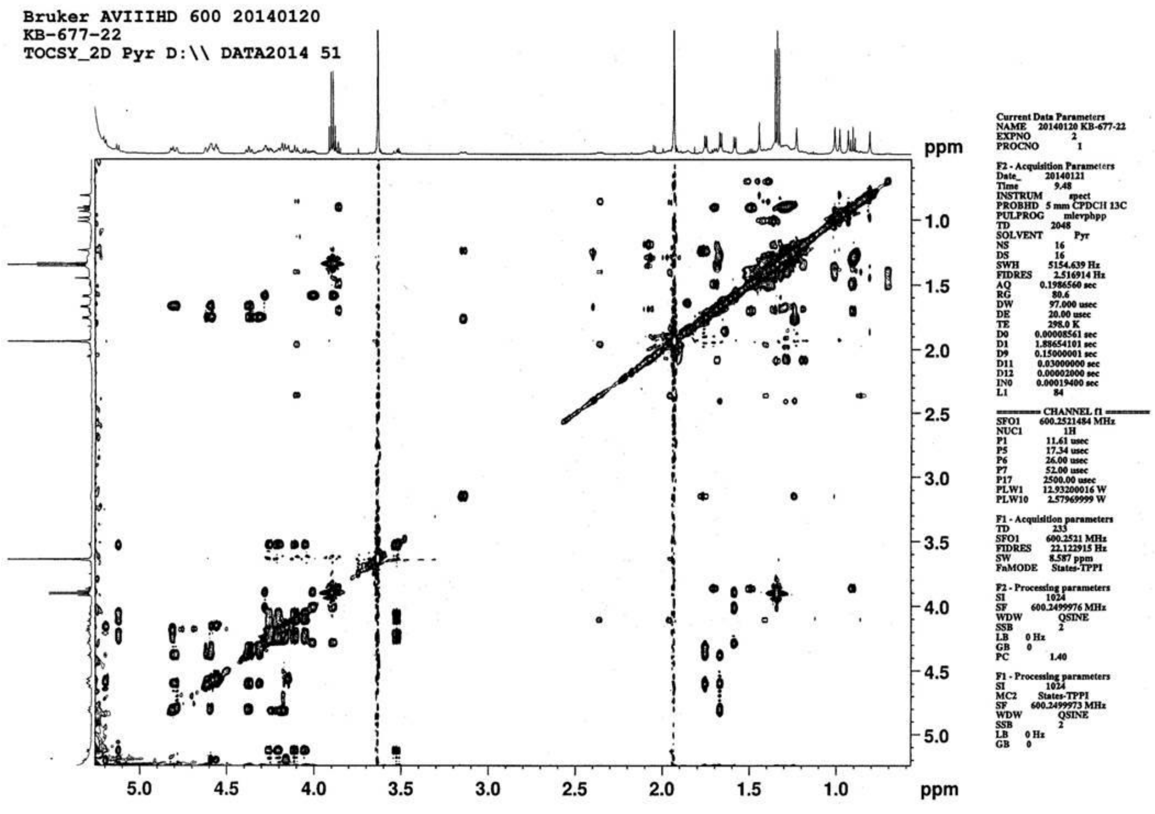


The part 3 of the TOCSY Spectrum of Compound C2.

**Figure S2.** *Cont.*

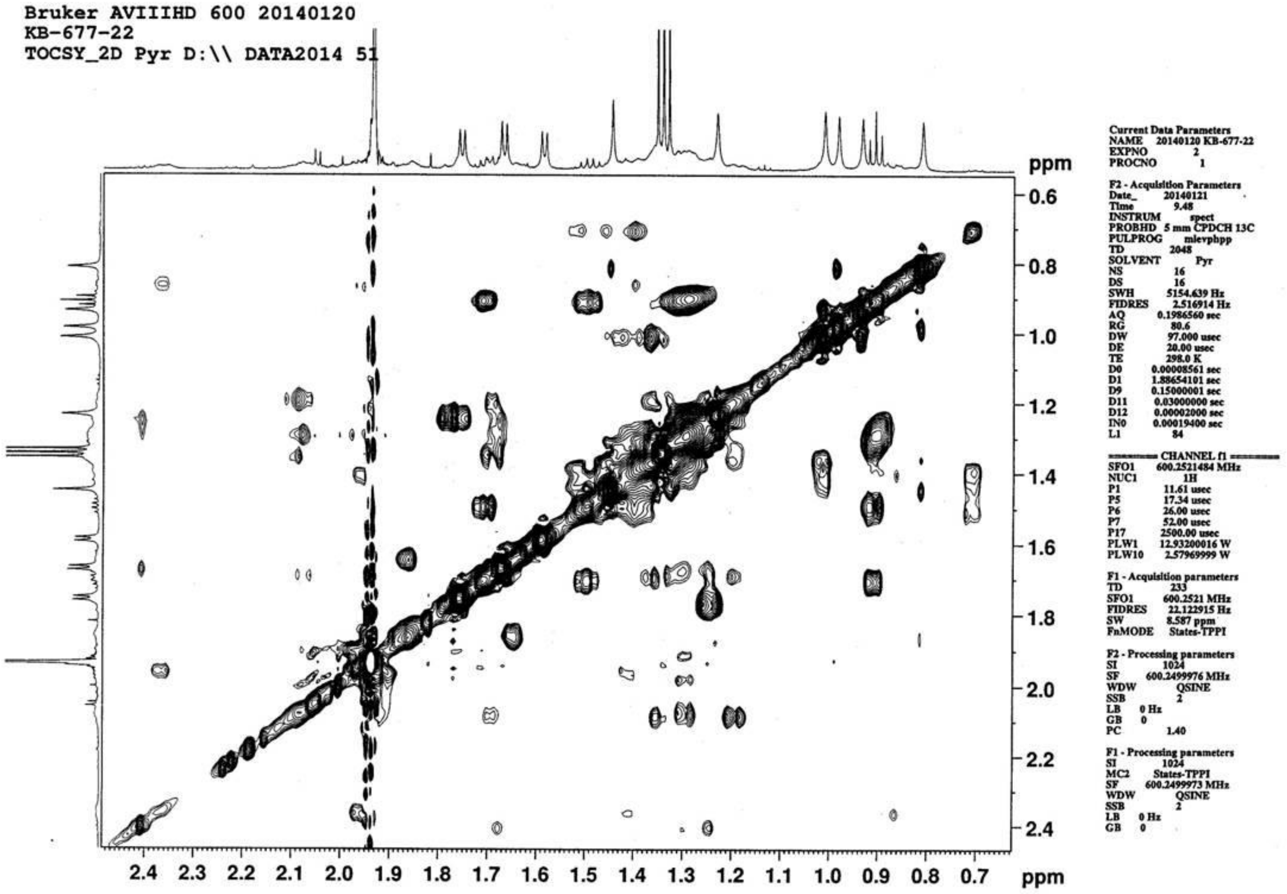


The part 4 of the TOCSY Spectrum of Compound C2.

**Figure S3.** The HPLC Chromatography of the derivatives of sugars in Compound **C1**.

```
Data file   : D:\DATA\JTF\A 2013-06-05 10-43-11\041-0101.D
Sample Name: 2-1                                                    1
==========================================================================
Injection Date   : Wed, 5. Jun. 2013              Seq Line   :        1
Sample Name      : 2-1                            Location   :  Vial 41
Acq Operator     : SLL                            Inj. No.   :        1
                                                  Inj. Vol.  :    20 µl

Acq. Method      : D:\DATA\JTF\A 2013-06-05 10-43-11\JTF.M
Analysis Method : C:\CHEM32\1\METHODS\DEF_LC.M
Last Changed     : Wed, 26. Jun. 2013, 10:02:45 am
                  (modified after loading)
```

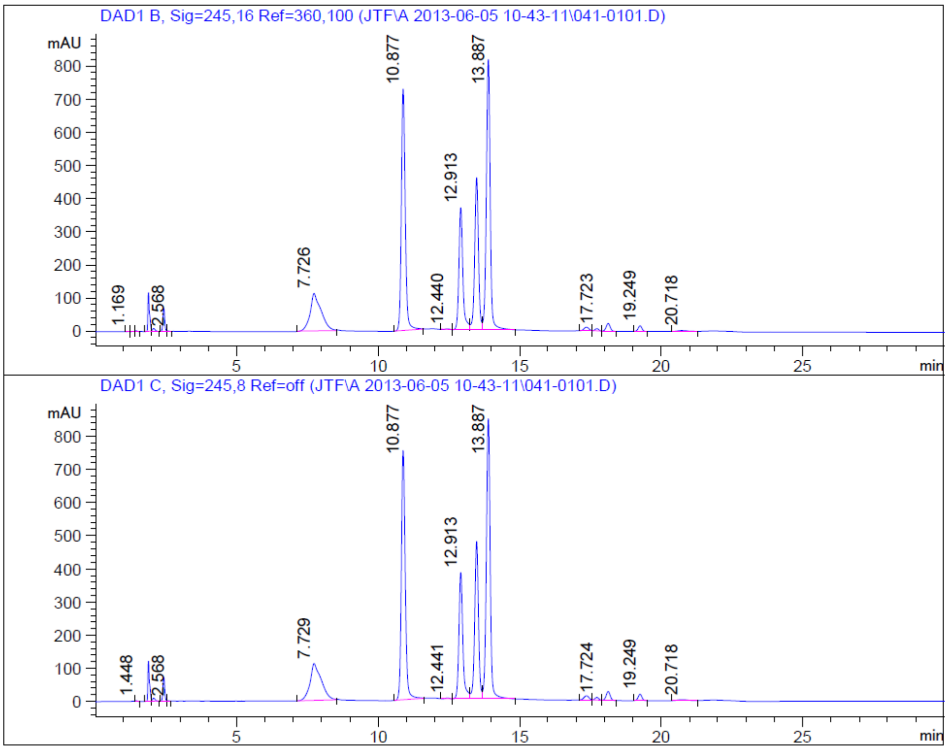


```
==========================================================================
                Customized Report: Area Pecent Report
==========================================================================


Multiplier               :  1.000000
Dilution                 :  1.000000
Uncalibrated Peaks       :  not reported

Signal  1: DAD1 B, Sig=245,16 Ref=360,100
```

| Peak # | RT [min] | Type | Width [min] | Height [mAU] | Area % | Area [mAU*s] |
|---|---|---|---|---|---|---|
| 1 | 1.169 | BV | 0.087 | 1.003 | 0.021 | 5.732 |
| 2 | 1.323 | VV | 0.123 | 1.387 | 0.037 | 10.096 |
| 3 | 1.446 | VB | 0.060 | 3.271 | 0.050 | 13.484 |
| 4 | 1.878 | BV | 0.058 | 118.094 | 1.716 | 466.661 |
| 5 | 2.060 | VB | 0.102 | 9.423 | 0.231 | 62.857 |

**Figure S3.** *Cont.*

Data file : D:\DATA\JTF\A 2013-06-05 10-43-11\041-0101.D
Sample Name: 2-1 2

| Peak # | RT [min] | Type | Width [min] | Height [mAU] | Area % % | Area [mAU*s] |
|---|---|---|---|---|---|---|
| 6 | 2.418 | BV | 0.071 | 69.837 | 1.169 | 317.954 |
| 7 | 2.568 | VV | 0.068 | 1.576 | 0.027 | 7.345 |
| 8 | 7.726 | BB | 0.355 | 113.697 | 11.299 | 3073.369 |
| 9 | 10.877 | BB | 0.145 | 729.668 | 25.593 | 6961.459 |
| 10 | 12.440 | BV | 0.181 | 2.074 | 0.082 | 22.252 |
| 11 | 12.913 | VV | 0.148 | 368.248 | 13.189 | 3587.517 |
| 12 | 13.473 | VV | 0.142 | 458.987 | 15.631 | 4251.577 |
| 13 | 13.887 | VB | 0.145 | 816.262 | 28.463 | 7742.103 |
| 14 | 17.354 | BV | 0.202 | 11.466 | 0.549 | 149.386 |
| 15 | 17.723 | VV | 0.152 | 7.526 | 0.270 | 73.446 |
| 16 | 18.122 | VB | 0.157 | 23.426 | 0.865 | 235.264 |
| 17 | 19.249 | BB | 0.145 | 16.865 | 0.582 | 158.271 |
| 18 | 20.718 | BB | 0.339 | 2.641 | 0.226 | 61.422 |

Signal 2: DAD1 C, Sig=245,8 Ref=off

| Peak # | RT [min] | Type | Width [min] | Height [mAU] | Area % % | Area [mAU*s] |
|---|---|---|---|---|---|---|
| 1 | 1.448 | VB | 0.063 | 2.545 | 0.038 | 10.583 |
| 2 | 1.878 | BV | 0.058 | 122.080 | 1.715 | 482.782 |
| 3 | 2.060 | VB | 0.102 | 9.639 | 0.229 | 64.497 |
| 4 | 2.418 | BV | 0.071 | 72.319 | 1.171 | 329.658 |
| 5 | 2.568 | VV | 0.069 | 1.667 | 0.028 | 7.841 |
| 6 | 7.729 | BB | 0.364 | 111.709 | 11.031 | 3104.410 |
| 7 | 10.877 | BB | 0.145 | 753.384 | 25.550 | 7190.292 |
| 8 | 12.441 | BV | 0.181 | 2.149 | 0.082 | 22.952 |
| 9 | 12.913 | VV | 0.148 | 381.733 | 13.215 | 3719.042 |
| 10 | 13.473 | VV | 0.142 | 476.033 | 15.669 | 4409.695 |
| 11 | 13.887 | VB | 0.145 | 846.198 | 28.518 | 8025.639 |
| 12 | 17.354 | BV | 0.202 | 13.212 | 0.613 | 172.390 |
| 13 | 17.724 | VV | 0.152 | 8.679 | 0.302 | 85.074 |
| 14 | 18.122 | VB | 0.157 | 26.960 | 0.964 | 271.164 |
| 15 | 19.249 | BB | 0.145 | 19.364 | 0.646 | 181.728 |
| 16 | 20.718 | BB | 0.342 | 2.770 | 0.229 | 64.583 |

**Figure S4.** The HPLC Chromatography of the derivatives of sugars in Compound **C2**.

```
Data file  : D:\DATA\JTF\A 2013-06-05 10-43-11\042-0201.D
Sample Name: 2-2                                                     1
=====================================================================
Injection Date  : Wed, 5. Jun. 2013              Seq Line   :        2
Sample Name     : 2-2                            Location   :  Vial 42
Acq Operator    : SLL                            Inj. No.   :        1
                                                 Inj. Vol.  :    20 µl

Acq. Method     : D:\DATA\JTF\A 2013-06-05 10-43-11\JTF.M
Analysis Method : C:\CHEM32\1\METHODS\DEF_LC.M
Last Changed    : Wed, 26. Jun. 2013, 10:02:45 am
                  (modified after loading)
```

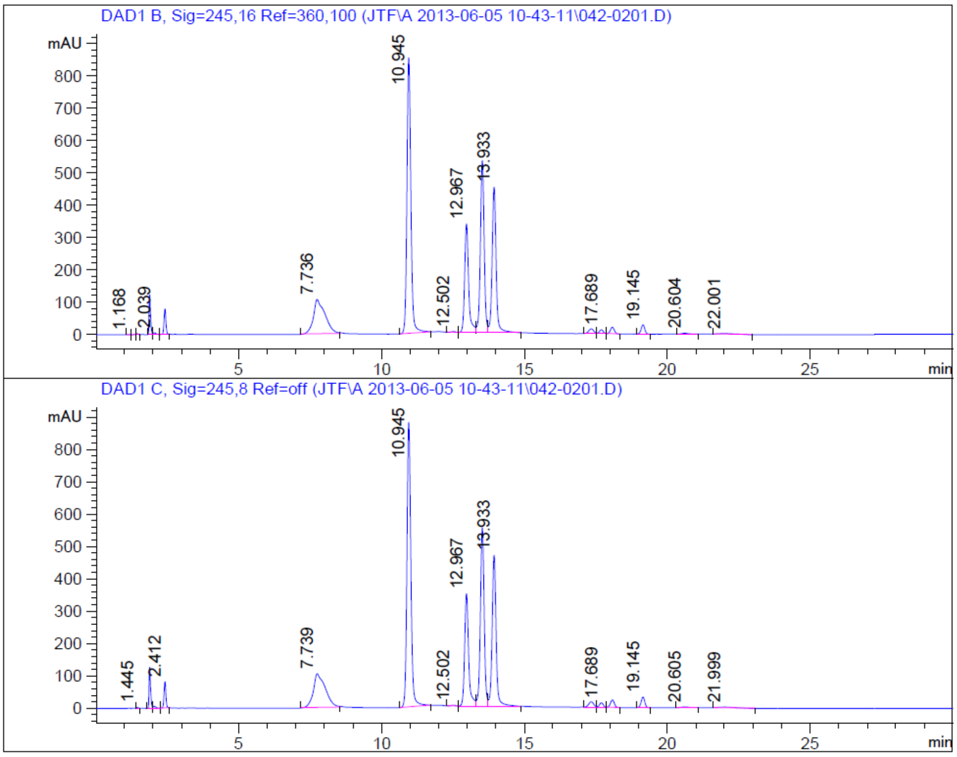


```
=====================================================================
              Customized Report: Area Pecent Report
=====================================================================

Multiplier                : 1.000000
Dilution                  : 1.000000
Uncalibrated Peaks        : not reported

Signal   1: DAD1 B, Sig=245,16 Ref=360,100
```

| Peak # | RT [min] | Type | Width [min] | Height [mAU] | Area % % | Area [mAU*s] |
|---|---|---|---|---|---|---|
| 1 | 1.168 | BV | 0.091 | 1.007 | 0.023 | 5.915 |
| 2 | 1.324 | VV | 0.121 | 1.359 | 0.038 | 9.656 |
| 3 | 1.445 | VB | 0.052 | 3.670 | 0.049 | 12.523 |
| 4 | 1.876 | BV | 0.054 | 123.814 | 1.748 | 447.414 |
| 5 | 2.039 | VV | 0.100 | 5.760 | 0.151 | 38.756 |

**Figure S4.** *Cont.*

Data file : D:\DATA\JTF\A 2013-06-05 10-43-11\042-0201.D
Sample Name: 2-2 2

| Peak # | RT [min] | Type | Width [min] | Height [mAU] | Area % % | Area [mAU*s] |
|---|---|---|---|---|---|---|
| 6 | 2.412 | VB | 0.072 | 79.832 | 1.441 | 368.717 |
| 7 | 7.736 | BB | 0.372 | 107.071 | 11.911 | 3048.418 |
| 8 | 10.945 | BB | 0.145 | 853.646 | 31.735 | 8121.967 |
| 9 | 12.502 | BV | 0.178 | 2.347 | 0.096 | 24.529 |
| 10 | 12.967 | VV | 0.147 | 336.161 | 12.759 | 3265.373 |
| 11 | 13.520 | VV | 0.142 | 533.083 | 19.285 | 4935.545 |
| 12 | 13.933 | VB | 0.148 | 450.831 | 17.165 | 4393.049 |
| 13 | 17.334 | BV | 0.198 | 14.933 | 0.742 | 189.782 |
| 14 | 17.689 | VV | 0.149 | 12.426 | 0.470 | 120.365 |
| 15 | 18.073 | VB | 0.152 | 20.601 | 0.785 | 200.893 |
| 16 | 19.145 | BB | 0.139 | 28.793 | 1.010 | 258.557 |
| 17 | 20.604 | BB | 0.288 | 2.431 | 0.178 | 45.489 |
| 18 | 22.001 | BB | 0.585 | 2.428 | 0.415 | 106.263 |

Signal 2: DAD1 C, Sig=245,8 Ref=off

| Peak # | RT [min] | Type | Width [min] | Height [mAU] | Area % % | Area [mAU*s] |
|---|---|---|---|---|---|---|
| 1 | 1.445 | VB | 0.053 | 2.854 | 0.038 | 10.023 |
| 2 | 1.876 | BV | 0.054 | 127.741 | 1.740 | 460.700 |
| 3 | 2.039 | VB | 0.099 | 5.739 | 0.142 | 37.649 |
| 4 | 2.412 | BB | 0.071 | 82.432 | 1.427 | 377.799 |
| 5 | 7.739 | BB | 0.381 | 105.302 | 11.634 | 3080.844 |
| 6 | 10.945 | BB | 0.145 | 881.365 | 31.675 | 8388.284 |
| 7 | 12.502 | BV | 0.178 | 2.442 | 0.096 | 25.537 |
| 8 | 12.967 | VV | 0.147 | 348.442 | 12.782 | 3384.880 |
| 9 | 13.520 | VV | 0.142 | 552.816 | 19.327 | 5118.388 |
| 10 | 13.933 | VB | 0.148 | 467.379 | 17.192 | 4552.745 |
| 11 | 17.334 | BV | 0.198 | 17.191 | 0.826 | 218.670 |
| 12 | 17.689 | VV | 0.149 | 14.314 | 0.525 | 138.917 |
| 13 | 18.073 | VB | 0.152 | 23.710 | 0.874 | 231.424 |
| 14 | 19.145 | BB | 0.139 | 33.072 | 1.122 | 297.064 |
| 15 | 20.605 | BB | 0.289 | 2.556 | 0.181 | 48.030 |
| 16 | 21.999 | BB | 0.581 | 2.549 | 0.421 | 111.551 |

**Figure S5.** The HPLC Chromatography of the derivative of L-Rhamnose.

```
Data file   : D:\DATA\JTF\A 2013-06-04 16-25-07\035-0501.D
Sample Name: 2-R                                                         1
===========================================================================
Injection Date  : Tue, 4. Jun. 2013                Seq Line   :          5
Sample Name     : 2-R                              Location   :    Vial 35
Acq Operator    : SLL                              Inj. No.   :          1
                                                   Inj. Vol.  :      20 µl

Acq. Method     : D:\DATA\JTF\A 2013-06-04 16-25-07\JTF.M
Analysis Method : C:\CHEM32\1\METHODS\DEF_LC.M
Last Changed    : Wed, 26. Jun. 2013, 10:02:45 am
                  (modified after loading)
```

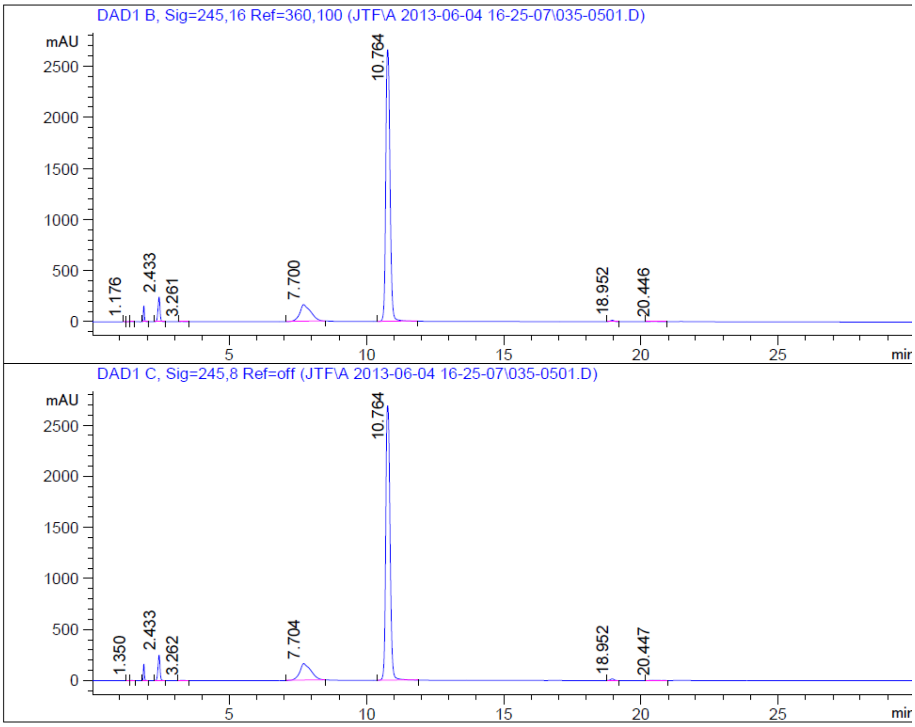


```
===========================================================================
                     Customized Report: Area Pecent Report
===========================================================================


Multiplier                : 1.000000
Dilution                  : 1.000000
Uncalibrated Peaks        : not reported

Signal  1: DAD1 B, Sig=245,16 Ref=360,100
```

| Peak # | RT [min] | Type | Width [min] | Height [mAU] | Area % % | Area [mAU*s] |
|---|---|---|---|---|---|---|
| 1 | 1.176 | BV | 0.070 | 1.195 | 0.015 | 5.326 |
| 2 | 1.350 | VV | 0.125 | 1.718 | 0.036 | 12.737 |
| 3 | 1.434 | VB | 0.055 | 5.449 | 0.058 | 20.209 |
| 4 | 1.876 | BB | 0.044 | 154.442 | 1.198 | 418.829 |
| 5 | 2.433 | BB | 0.077 | 241.809 | 3.498 | 1222.512 |

**Figure S5.** *Cont.*

Data file : D:\DATA\JTF\A 2013-06-04 16-25-07\035-0501.D
Sample Name: 2-R 2

| Peak # | RT [min] | Type | Width [min] | Height [mAU] | Area % % | Area [mAU*s] |
|---|---|---|---|---|---|---|
| 6 | 3.261 | BB | 0.131 | 1.627 | 0.043 | 15.170 |
| 7 | 7.700 | BB | 0.376 | 165.692 | 13.422 | 4691.088 |
| 8 | 10.764 | BB | 0.166 | 2662.881 | 81.242 | 28394.283 |
| 9 | 18.952 | BB | 0.141 | 14.090 | 0.371 | 129.772 |
| 10 | 20.446 | BB | 0.295 | 2.171 | 0.116 | 40.398 |

Signal 2: DAD1 C, Sig=245,8 Ref=off

| Peak # | RT [min] | Type | Width [min] | Height [mAU] | Area % % | Area [mAU*s] |
|---|---|---|---|---|---|---|
| 1 | 1.350 | VV | 0.104 | 1.204 | 0.024 | 8.706 |
| 2 | 1.433 | VB | 0.056 | 4.256 | 0.045 | 16.143 |
| 3 | 1.876 | BB | 0.044 | 160.668 | 1.218 | 435.131 |
| 4 | 2.433 | BB | 0.077 | 250.172 | 3.540 | 1264.697 |
| 5 | 3.262 | BB | 0.135 | 1.726 | 0.047 | 16.728 |
| 6 | 7.704 | BB | 0.384 | 163.681 | 13.276 | 4742.915 |
| 7 | 10.764 | BB | 0.170 | 2690.479 | 81.312 | 29048.945 |
| 8 | 18.952 | BB | 0.141 | 16.177 | 0.417 | 148.868 |
| 9 | 20.447 | BB | 0.299 | 2.291 | 0.120 | 42.999 |

**Figure S6.** The HPLC Chromatography of the derivative of D-Glucose.

Data file : D:\DATA\JTF\A 2013-06-04 16-25-07\033-0301.D
Sample Name: 2-G 1

Injection Date : Tue, 4. Jun. 2013 Seq Line : 3
Sample Name : 2-G Location : Vial 33
Acq Operator : SLL Inj. No. : 1
Inj. Vol. : 20 µl

Acq. Method : D:\DATA\JTF\A 2013-06-04 16-25-07\JTF.M
Analysis Method : C:\CHEM32\1\METHODS\DEF_LC.M
Last Changed : Wed, 26. Jun. 2013, 10:02:45 am
(modified after loading)

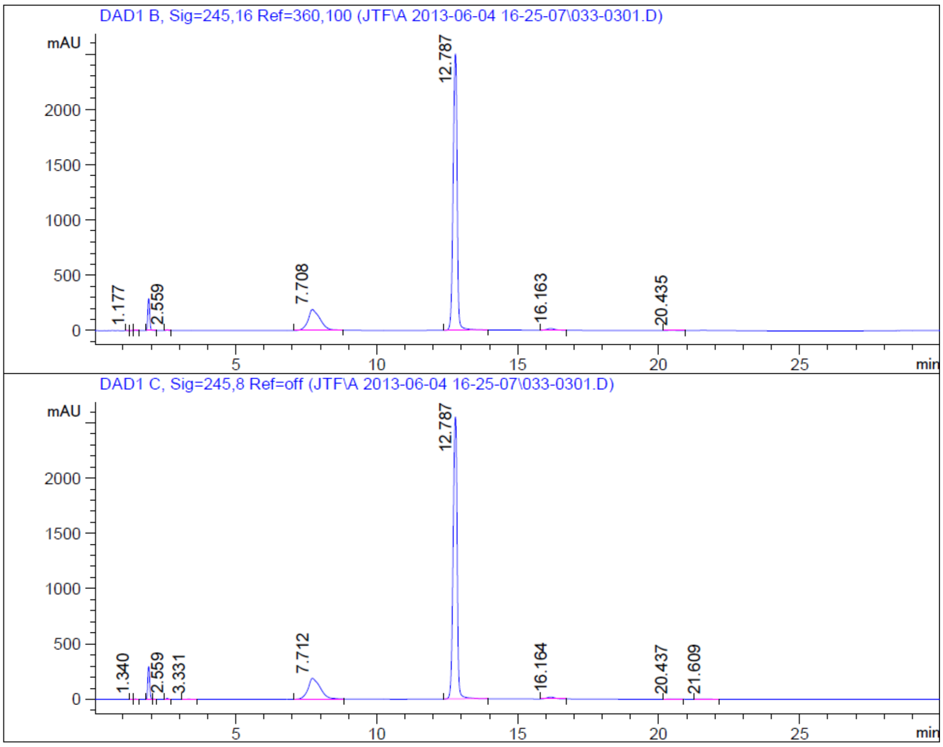


Customized Report: Area Pecent Report

Multiplier : 1.000000
Dilution : 1.000000
Uncalibrated Peaks : not reported

Signal 1: DAD1 B, Sig=245,16 Ref=360,100

| Peak # | RT [min] | Type | Width [min] | Height [mAU] | Area % % | Area [mAU*s] |
|---|---|---|---|---|---|---|
| 1 | 1.177 | BV | 0.074 | 1.248 | 0.019 | 6.008 |
| 2 | 1.324 | VV | 0.109 | 1.732 | 0.038 | 12.357 |
| 3 | 1.433 | VB | 0.055 | 5.841 | 0.066 | 21.424 |
| 4 | 1.904 | BB | 0.081 | 288.180 | 4.335 | 1397.411 |
| 5 | 2.559 | BB | 0.070 | 8.068 | 0.112 | 36.085 |

Instrument 1 Fri, 14. Feb. 2014 11:21:42 am Page 1 of 2

**Figure S6.** *Cont.*

Data file : D:\DATA\JTF\A 2013-06-04 16-25-07\033-0301.D
Sample Name: 2-G 2

| Peak # | RT [min] | Type | Width [min] | Height [mAU] | Area % % | Area [mAU*s] |
|---|---|---|---|---|---|---|
| 6 | 7.708 | BB | 0.392 | 190.515 | 17.425 | 5616.439 |
| 7 | 12.787 | BB | 0.156 | 2500.485 | 76.946 | 24801.738 |
| 8 | 16.163 | BB | 0.306 | 15.031 | 0.920 | 296.519 |
| 9 | 20.435 | BB | 0.306 | 2.268 | 0.139 | 44.764 |

Signal 2: DAD1 C, Sig=245,8 Ref=off

| Peak # | RT [min] | Type | Width [min] | Height [mAU] | Area % % | Area [mAU*s] |
|---|---|---|---|---|---|---|
| 1 | 1.340 | VV | 0.108 | 1.220 | 0.028 | 9.154 |
| 2 | 1.432 | VB | 0.060 | 4.788 | 0.059 | 19.605 |
| 3 | 1.904 | BV | 0.080 | 296.711 | 4.322 | 1433.953 |
| 4 | 2.075 | VB | 0.061 | 2.119 | 0.026 | 8.583 |
| 5 | 2.559 | BB | 0.070 | 8.326 | 0.112 | 37.311 |
| 6 | 3.331 | BB | 0.177 | 1.109 | 0.039 | 13.028 |
| 7 | 7.712 | BB | 0.401 | 189.382 | 17.191 | 5703.253 |
| 8 | 12.787 | BB | 0.159 | 2551.865 | 76.942 | 25526.463 |
| 9 | 16.164 | BB | 0.306 | 17.410 | 1.041 | 345.519 |
| 10 | 20.437 | BB | 0.260 | 2.348 | 0.131 | 43.430 |
| 11 | 21.609 | BB | 0.338 | 1.448 | 0.108 | 35.778 |

**Figure S7.** The HPLC Chromatography of the derivative of D-Fructose.

Data file : D:\DATA\JTF\A 2013-06-04 16-25-07\034-0401.D
Sample Name: 2-F 1

=====================================================================

Injection Date : Tue, 4. Jun. 2013 Seq Line : 4
Sample Name : 2-F Location : Vial 34
Acq Operator : SLL Inj. No. : 1
Inj. Vol. : 20 µl

Acq. Method : D:\DATA\JTF\A 2013-06-04 16-25-07\JTF.M
Analysis Method : C:\CHEM32\1\METHODS\DEF_LC.M
Last Changed : Wed, 26. Jun. 2013, 10:02:45 am
(modified after loading)

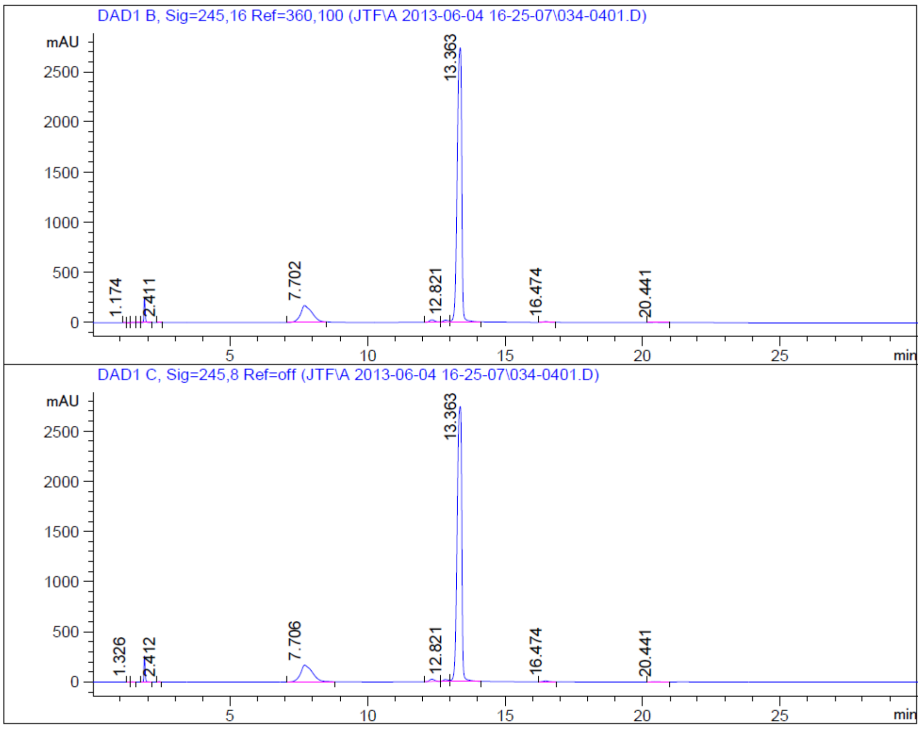


=====================================================================

Customized Report: Area Pecent Report

=====================================================================

Multiplier : 1.000000
Dilution : 1.000000
Uncalibrated Peaks : not reported

Signal 1: DAD1 B, Sig=245,16 Ref=360,100

| Peak # | RT [min] | Type | Width [min] | Height [mAU] | Area % | Area [mAU*s] |
|---|---|---|---|---|---|---|
| 1 | 1.174 | BV | 0.075 | 1.480 | 0.019 | 7.263 |
| 2 | 1.321 | VV | 0.105 | 2.067 | 0.038 | 14.418 |
| 3 | 1.437 | VB | 0.068 | 5.063 | 0.064 | 24.098 |
| 4 | 1.649 | BV | 0.127 | 1.400 | 0.033 | 12.350 |
| 5 | 1.880 | VB | 0.048 | 253.648 | 2.051 | 775.399 |

**Figure S7.** *Cont.*

Data file : D:\DATA\JTF\A 2013-06-04 16-25-07\034-0401.D
Sample Name: 2-F 2

| Peak # | RT [min] | Type | Width [min] | Height [mAU] | Area % % | Area [mAU*s] |
|---|---|---|---|---|---|---|
| 6 | 2.411 | BB | 0.067 | 5.047 | 0.059 | 22.213 |
| 7 | 7.702 | BB | 0.389 | 168.612 | 13.038 | 4928.301 |
| 8 | 12.334 | BV | 0.223 | 24.305 | 0.922 | 348.687 |
| 9 | 12.821 | VV | 0.194 | 20.983 | 0.772 | 291.965 |
| 10 | 13.363 | VB | 0.183 | 2734.718 | 82.597 | 31221.105 |
| 11 | 16.474 | BB | 0.218 | 7.717 | 0.301 | 113.628 |
| 12 | 20.441 | BB | 0.296 | 2.159 | 0.106 | 39.998 |

Signal 2: DAD1 C, Sig=245,8 Ref=off

| Peak # | RT [min] | Type | Width [min] | Height [mAU] | Area % % | Area [mAU*s] |
|---|---|---|---|---|---|---|
| 1 | 1.326 | VV | 0.105 | 1.267 | 0.023 | 8.993 |
| 2 | 1.437 | VB | 0.067 | 3.597 | 0.044 | 16.957 |
| 3 | 1.880 | VB | 0.047 | 262.097 | 2.044 | 789.036 |
| 4 | 2.412 | BB | 0.063 | 4.973 | 0.052 | 20.004 |
| 5 | 7.706 | BB | 0.402 | 167.927 | 13.180 | 5088.791 |
| 6 | 12.334 | BV | 0.223 | 25.181 | 0.934 | 360.697 |
| 7 | 12.821 | VV | 0.194 | 21.671 | 0.779 | 300.808 |
| 8 | 13.363 | VB | 0.187 | 2742.731 | 82.498 | 31853.342 |
| 9 | 16.474 | BB | 0.219 | 8.890 | 0.337 | 130.097 |
| 10 | 20.441 | BB | 0.296 | 2.281 | 0.109 | 42.274 |

**Figure S8.** The HPLC Chromatography of the derivative of D-Xylose.

```
Data file   : D:\DATA\JTF\A 2013-06-04 16-25-07\036-0601.D
Sample Name: 2-X                                                      1
=====================================================================
Injection Date  : Tue, 4. Jun. 2013                Seq Line    :       6
Sample Name     : 2-X                              Location    :  Vial 36
Acq Operator    : SLL                              Inj. No.    :       1
                                                   Inj. Vol.   :    20 µl

Acq. Method     : D:\DATA\JTF\A 2013-06-04 16-25-07\JTF.M
Analysis Method : C:\CHEM32\1\METHODS\DEF_LC.M
Last Changed    : Wed, 26. Jun. 2013, 10:02:45 am
                  (modified after loading)
```

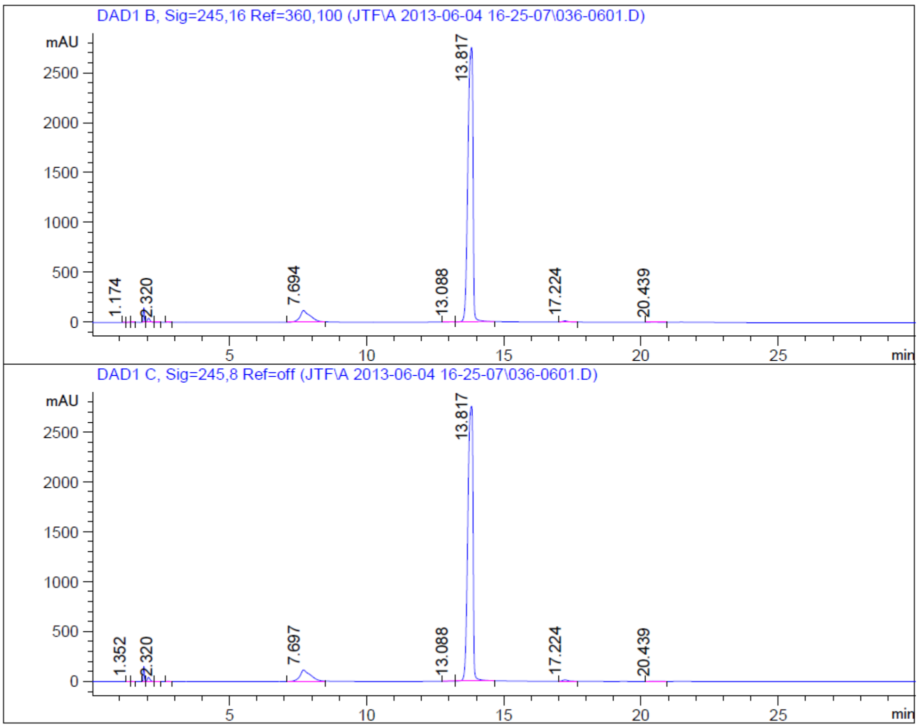


```
=====================================================================
           Customized Report: Area Pecent Report
=====================================================================

Multiplier              :  1.000000
Dilution                :  1.000000
Uncalibrated Peaks      :  not reported

Signal  1: DAD1 B, Sig=245,16 Ref=360,100
```

| Peak # | RT [min] | Type | Width [min] | Height [mAU] | Area % | Area [mAU*s] |
|---|---|---|---|---|---|---|
| 1 | 1.174 | BV | 0.075 | 1.172 | 0.014 | 5.560 |
| 2 | 1.352 | VV | 0.123 | 1.629 | 0.030 | 11.868 |
| 3 | 1.434 | VB | 0.056 | 5.276 | 0.050 | 19.765 |
| 4 | 1.874 | BV | 0.044 | 148.290 | 1.026 | 401.687 |
| 5 | 2.042 | VV | 0.102 | 41.796 | 0.718 | 280.888 |

**Figure S8.** *Cont.*

Data file : D:\DATA\JTF\A 2013-06-04 16-25-07\036-0601.D
Sample Name: 2-X 2

| Peak # | RT [min] | Type | Width [min] | Height [mAU] | Area % % | Area [mAU*s] |
|---|---|---|---|---|---|---|
| 6 | 2.320 | VB | 0.095 | 1.248 | 0.022 | 8.482 |
| 7 | 2.751 | BB | 0.083 | 2.871 | 0.040 | 15.614 |
| 8 | 7.694 | BB | 0.349 | 117.653 | 7.858 | 3075.641 |
| 9 | 13.088 | BV | 0.200 | 1.247 | 0.042 | 16.467 |
| 10 | 13.817 | VB | 0.209 | 2749.947 | 89.662 | 35092.391 |
| 11 | 17.224 | BB | 0.209 | 12.134 | 0.434 | 169.819 |
| 12 | 20.439 | BB | 0.290 | 2.197 | 0.103 | 40.351 |

Signal 2: DAD1 C, Sig=245,8 Ref=off

| Peak # | RT [min] | Type | Width [min] | Height [mAU] | Area % % | Area [mAU*s] |
|---|---|---|---|---|---|---|
| 1 | 1.352 | VV | 0.104 | 1.131 | 0.020 | 7.982 |
| 2 | 1.434 | VB | 0.056 | 4.140 | 0.040 | 15.773 |
| 3 | 1.874 | BV | 0.044 | 154.247 | 1.047 | 417.318 |
| 4 | 2.042 | VV | 0.102 | 42.816 | 0.721 | 287.495 |
| 5 | 2.320 | VB | 0.095 | 1.282 | 0.022 | 8.736 |
| 6 | 2.751 | BB | 0.083 | 2.950 | 0.040 | 16.003 |
| 7 | 7.697 | BB | 0.358 | 114.655 | 7.751 | 3089.331 |
| 8 | 13.088 | BV | 0.197 | 1.252 | 0.041 | 16.230 |
| 9 | 13.817 | VB | 0.212 | 2753.128 | 89.717 | 35760.539 |
| 10 | 17.224 | BB | 0.210 | 14.041 | 0.496 | 197.565 |
| 11 | 20.439 | BB | 0.291 | 2.312 | 0.106 | 42.217 |
